# How Early Life Influences Shape Pathways to Longevity: Insights From the Georgia Centenarian Study

**DOI:** 10.1093/geroni/igaf122.066

**Published:** 2025-12-31

**Authors:** Grace da Rosa, Amin Hashemzehi, Peter Martin, Leonard Poon

**Affiliations:** Georgia State University, Atlanta, Georgia, United States; Iowa State University, Ames, Iowa, United States; Iowa State University, Ames, Iowa, United States; University of Georgia, Athens, Georgia, United States

## Abstract

Understanding early life influences is crucial for uncovering pathways to health and longevity, especially in those who live to 100 and beyond. This research examines early life conditions of centenarians and their potential influence on the pathways to longevity, focusing on 239 participants aged 98 to 109 years from the Georgia Centenarian Study. A latent profile analysis (LPA) identified two distinct groups. Group 1 (n = 90, 38.58%, Early Disadvantaged Centenarians) is characterized by lower parental and centenarian education, parents with shorter life span, poorer health during childhood, and lower financial well-being in childhood. In contrast, Group 2 (n = 142, 61.42%, Early Resourceful Centenarians) had parents with higher education levels and greater longevity, participants’ higher education, and greater financial well-being during childhood compared to Group 1. T-tests and Chi-Square results confirmed significant differences with more favorable education of parents and of participants in Group 2. This group also reported growing up in a financially favorably household and their fathers and mothers lived significantly longer than their counterparts in Group 1. Group 1 had a larger percentage of African American participants and participants had significantly more children when compared to Group 2. ADL functioning also differed significantly, with Group 1 exhibiting lower ADL scores. These findings highlight the importance of early-life factors in shaping pathways to longevity and quality of life in centenarians.

